# Exploring the relationship between burnout and emotional intelligence among academics and clinicians at King Saud University

**DOI:** 10.1186/s12909-023-04604-7

**Published:** 2023-09-18

**Authors:** Aljohara S. Almeneessier, Samy A. Azer

**Affiliations:** 1 Family and Community Medicine Department, College of Medicine, King Saud University, Riyadh, Saudi Arabia; 2 King Saud University Medical City, King Saud University, Riyadh, Kingdom of Saudi Arabia; 3 Medical Education Department, College of Medicine, King Saud University, P O Box 2925, Riyadh, 11461 Saudi Arabia

**Keywords:** Burnout, Emotional intelligence, Academics, Clinicians

## Abstract

**Background:**

Academics and clinicians are exposed to significant workload pressures and are at a high risk of stress and burnout.

**Objectives:**

This study aimed to examine the relationship between burnout and emotional intelligence (EI) by comparing and corelating burnout and EI scores among academics and clinicians against several factors.

**Methods:**

In this cross-sectional study, academics and clinicians at King Saud University and King Saud University Medical City and Affiliated Hospitals were invited to complete anonymous questionnaires: Maslach Burnout Inventory-Human Services Survey and the Trait Emotional Intelligence Questionnaire Short Form. The collected data were analyzed using the SPSS software for descriptive studies, group comparisons, regression analyses, and Pearson's (r) correlation tests.

**Results:**

Study participants included 126 individuals (men = 65, 51.6%; women = 61, 48.4%). Of these, 65% were Saudi nationals and 35% were expatriates, and 76 were academics while 50 were clinicians. The mean (minimum to maximum) burnout total score was 55 ± 18.9 (8 to 97) and the global TEIQue-SF score ranged between 2.8 and 6.7 (5.04 ± 0.7). Burnout scores varied between departments and were higher among younger participants and non-Saudis. Age had a small direct correlation with self-control (*r* = .17, *p* = .05), and there was no statistically significant correlation with other EI factors. However, there was a moderate inverse correlation between age and emotional exhaustion (EE) (*r* = -0.33, *p* < 0.0001), and a small inverse correlation with depersonalization (DP) (*r* = -0.21, *p* = 0.02). T-tests demonstrated a statistically significant difference in EI factor "emotionality" among Saudis (5.2 ± .8) and non-Saudis (4.9 ± .8) (t124 = 2.2, *p* = 0.03), and for burnout subscales, there was a statistically significant difference in DP among Saudis (6.4 ± 4.8) and non-Saudis (8.5 ± 5.6), (*p* = 0.03). Moderate (*r* = -0.3, *p* = 0.01) and weak (*r* = -0.2, *p* = 0.05) negative correlations were found between EI factors and burnout subscales (EE, DP).

**Conclusion:**

This study confirmed an inverse relationship between burnout and EI scores among academics and clinicians. The findings suggest the need for introducing measures and implementing a system for early detection of burnout among staff and providing support to enhance EI and requisite care for those undergoing burnout episodes.

## Introduction

Burnout was first introduced in the 1970s to describe the gradual emotional depletion and loss of motivation. Herbert Freudenberger defined it as “a state of mental and physical exhaustion caused by one’s professional life” [1]. During the 80 s, Maslach and Jackson, based on their observations, defined burnout as “a syndrome characterized by emotional exhaustion, depersonalization, and lack of personal accomplishment” [2]. This definition is based on high scores for both emotional exhaustion (EE) and depersonalization (DP) and a low score for personal accomplishment (PA) [3]. The authors believed that it was not a somatic disease but an emotional change manifested by exhaustion, depersonalization, and diminished personal achievement. However, people with burnout may show symptoms such as depression, and treating depression in these people may improve the symptoms of burnout [4]. A cohort study of forty-six residents in internal medicine and surgery reported a direct relationship between stress burnout and symptomatology (mood disturbances, poor general health, and lower clinical competency) [5]. In another study among Dutch medical residents (*n* = 2115 out of 5126; response rate = 41%), 432 (20%) residents had burnout and 12% (of 2115) reported having suicidal thoughts with a higher prevalence in the burnout group (20% vs 7.6%) [6].

Medical academics and clinicians are professionals exposed to significant workload pressures. This may be due to the nature of their jobs, declining resources, and rising demands from students and patients. Consequently, they are more likely to experience stress or burnout than the general population [7, 8]. In the Middle East, a systematic review of 136 studies reported a 40–60% prevalence of burnout among healthcare workers. Forty-eight studies used Maslach Burnout Inventory (MBI) to study burnout among physicians and reported that EE scores ranged from 14.9 ± 7 to 32.3 ± 6 (scores 0–54), PD scores ranged from 2.8 ± 1.2 to 15.9 ± 3.9 (score 0–30), and PA scores ranged from 3.8 ± 1.1 to 34.9 ± 3.4 (score 0–48) with an overall prevalence among clinicians between 6.3% to 88.5% [9]. Globally the prevalence ranges between 0–89.9% as reported by a systematic review of data extracted from 182 studies from 45 countries. The burnout subscales measured by MBI-Human Services Survey (HSS) reported: EE scores between 25 and 30, DP scores between 3 and 13, and PA scores between 3 and 39 [10]. Another systematic review analyzing 19 studies on healthcare professionals reported a prevalence of burnout as high as 80% [11]. Several other studies examined healthcare staff wellbeing, burnout, and job performance [12, 13].

Emotional intelligence (EI) is a measure of how well an individual perceives and responds to their emotions and those of others [14]. In general, EI is responsible for 80% of life successes [15]. Therefore, for success in the medical profession, whether in patient care, teaching medical students, or conducting clinical research, physicians must recognize and respond appropriately to the emotions of patients and their families, students, colleagues, and healthcare staff working with them.

Burnout has been studied extensively in relation to EI to identify approaches to recognizing one’s liability for burnout, groups at risk, and best prevention strategies [16, 17]. Studies have shown that people with higher EI scores can deal with challenging situations through self-awareness and self-regulation, the two qualities required for a successful career in the medical profession and academia [18, 19]. Different strategic interventions targeting physician burnout, such as mindfulness, stress management, and small group discussions, have been reported to reduce physician burnout [20].

Therefore, this study aimed to (i) compare burnout and EI scores among academics and clinicians against several factors including age, gender, nationality, work duration, seniority in the hierarchy, specialty, and place of training after completing residency; (ii) examine the relationship between burnout and EI; and (iii) correlate EI scores with burnout. In this study we compared two participant groups: academics and clinicians. Based on our institute we define academics as medical staff with university titles who are appointed by King Saud University and their workload is nearly equally covering clinical healthcare services, teaching, and research. We define clinicians as physicians with clinical titles who are appointed by King Saud University Medical City and Affiliated Hospitals, and their main workload is patient healthcare with optional contribution to teaching and research.

## Methods

This was a cross-sectional study targeting academics (at King Saud University College of Medicine) and clinicians (at King Saud University Medical City and affiliated hospitals), in Riyadh, Saudi Arabia. To address the research aims we used the Maslach Burnout Inventory (MBI-HSS) to assess the three dimensions of burnout [21] and the Trait Emotional Intelligence Questionnaire Short Form (TEIQ-SF) [21, 22]. Although there are other tools available in the literature that have shown their potential, we decided to use MBI-HSS because it is widely used in research. Moreover, the National Academy of Medicine as per its website (https://nam.edu/valid-reliable-survey-instruments-measure-burnout-well-work-related-dimensions/) compared the different instruments on burnout and referred to MBI-HSS as the gold standard in research methods concerning burnout.

The study was approved by the King Saud University Institutional Review Board (IRB) and College of Medicine, IRB Number: E-19–3705, dated March 21, 2019.

The sample size was calculated according to the standard equation in the literature (Cochran’s equation: sample size = Z^2^pq/e^2^) [23]. A sample size of 273 participants was identified based on a 95% confidence level, a precision of 0.05, a prevalence of 80%, and a non-response rate of 10% (246/0.9 = 273) [24]. The targeted participants were academics and clinicians who had been on full-time contract and had joined this institute more than six months prior to the study. Participants were randomly selected by the faculty support unit and human resources department through emails.

The questionnaires used in this study were selected after reviewing the literature and other surveys used in RI and burnout research. The authors purchased a license to use the MBI-HSS (https://www.mindgarden.com/maslach-burnout-inventory-mbi/173-mbi-license-to-reproduce.html), a tool that can assess the three dimensions of burnout. It comprises 22 questions in the domains of emotional exhaustion (EE; 9 items), depersonalization (DP; 5 items), and personal accomplishment (PA; 8 items) [21, 25]. The reason for selecting this version rather than the abbreviated version (aMBI; 12 questions only) was the reported lack of concordance in burnout prevalence when the two surveys were used on a cohort of residents: 62.1% (aMBI) vs. 22.4% (MBI-HSS). This suggests an overestimation of burnout obtained from the shorter version and a higher yield of false-positive burnout estimations [26].

The Trait Emotional Intelligence Questionnaire Short Form (TEIQ-SF) comprises 30 questions, 2 from each of the 15 facets of the original questionnaire [22, 27, 28], which compromises 150 questions. The correlations between the short form (TEIQ-SF) factors and the full original form (TEIQ-FF) factors are strong, and range from *r* = 0.67 to r = 0.83 at *p* < 0.001; the unattenuated short form (TEIQ-SF) scales correlate strongly with the full form and range from *r* = 0.91 to *r* = 1.0 [29]. Hence, we chose the short form (TEIQ-SF) rather than the full form.

Before conducting the study, we ran a pilot study, with the aim to assess for any changes that were needed based on the feedback received, and assuring that the material prepared yields the outcomes planned for the study. The pilot comprised ten staff (five clinicians and five academics), not included in the final study. The participants were also asked about the clarity and comprehensibility of questions, and the time taken to complete the questionnaire.

The author (ASA) received a randomly selected list of emails (with help from the faculty support unit and human resources department). Participants were invited via email to complete the online anonymous questionnaire. The questionnaire used in the study comprised three parts: Part A: demographic data; Part B: the TEIQ-SF; and Part C: the MBI-HSS. The questionnaire was administered during the first academic semester, from September 2019 to February 2020. Participants were emailed every three weeks to remind them to complete the questionnaire. To increase the number of participants, twenty book vouchers each worth of SR 200 were made available to participants who joined in this survey; the winners were randomly selected by a third party not involved in the research, via a computer lottery program using an automated code number given to each completed questionnaire. The collected data were treated as highly confidential, and no information was identified or could expose anyone other than by the authors. All participants provided informed consent before starting the completion of the online questionnaire.

Maslach’s earlier MBI-HSS studies determined cut-off scores to categorize individuals under high, medium, or low burnout classification; Leiter and Maslach realized that the cut-off classification was invalid as a burnout diagnostic tool [21, 30] in addition to the reported variations in MBI cut-off scores among for different countries [31]. The MBI-HSS is scored on a 7-point Likert scale (0 = never) to 6 (every day). This follows the scoring keys for this questionnaire, and each subscale is scored separately and not combined to form an overall burnout score [21]. High scores on EE ≥ 27 (sum score 0–54) and DP ≥ 10 (sum score 0–30) are consistent with high burnout, while high scores on PA ≥ 40 (sum score 0–48) are associated with lower burnout [32].

The TEIQ-SF responses range from 1 = “Completely Disagree” to 7 = “Completely Agree”; the global trait score is calculated as the mean of the items’ scores; and the TEIQ-SF responses score between 1–7 [27].

The collected data were analyzed as means and standard deviations, and used to describe continuous variables; frequencies and percentages were used to describe categorical variables such as gender, years of employment, specialty, and nationality. Relative Importance Index Analysis (RII) was used as an item analysis to assess the relative weight [33]. The Bivariate Pearson's (r) test of correlation was used to assess the association between burnout, EI, and their subscales. The independent samples t-test and one-way analysis of variance (ANOVA) were used to assess the statistically significant mean differences between the groups. A multiple regression analysis was run to predict EE, DP, and PA based on gender, age, nationality, field of specialty, academic/clinical position, work duration, and Total TEIQ-EI scores. Alpha significance level was considered at 0.05 level.

## Results

A total of 126 individuals responded to the survey, with a response rate of 46.2%. Cronbach’s alpha for the study was 0.85 (TEIQue = 0.89 and MBI-HSS = 0.87). Participants’ mean age was 44 ± 10.2 years, with 51.6% men and 65% Saudi nationals. Table 1 shows the participants’ characteristics.

**Table 1:** Descriptive analysis of the participants' sociodemographic and professional characteristics. (*n* = 126)

Gender		
Male (n, %)	65	51.6
Female (n, %)	61	48.4
Age (Mean ± SD)	44 ± 10.2	
Nationality		
Saudi	82	65.1
Non-Saudi	44	34.9
Years of graduation (Mean ± SD)	19.8 ± 10.4	
Place of training after graduation (after MBBS)		
Local Program	74	58.7
Abroad Program	52	41.3
Specialty		
Family medicine	33	26.2
Medicine and Pediatric	30	23.8
Surgery and anesthesia	26	20.6
Basic science	18	14.3
Radiology	10	7.9
OB/Gyn	5	4
Dentists	4	3.2
Academic position		
Professor	21	16.7
Associate professor	20	15.9
Assistant professor	29	23
Lecturer/Demonstrator	6	4.8
Non-academic	50	39.7
Clinical position		
Consultant	75	59.5
Registrar/specialist	31	24.6
Residents	16	12.7
No clinical privilege	4	3.2
Length of employment		
≤ 5 years	38	30.2
6–10 years	25	19.8
11–15 years	22	17.5
16–20 years	10	7.9
More than 20 years	31	24.6
Holding an administrative position in addition to the current position		
Yes	37	29.4
No	89	70.6

The global TEIQue score for the participants ranged between 2.8 and 6.7 (5.04 ± 0.7). Burnout MBI-HSS overall mean ± SD score was 55 ± 18.9 (ranging from 8 to 97). The MBI subscales means were as follows: EE score was 19.5 ± 11, DP score was 7 ± 5.2, and PA score was 28.6 ± 9.4. Table 2 presents the descriptive analysis of the TEIQue global score and its four factors: emotionality, self-control, well-being, sociability and burnout subscales.

**Table 2:** Descriptive analysis of EI and its factors and burnout subscales

		TEIQue global score	Well-being	Self-control	emotionality	sociability	EE	DP	PA
Mean		5.0	5.3	4.8	5.0	4.7	19.5	7.0	28.6
Std. Deviation		.7	.9	.9	.8	.8	11.0	5.2	9.4
Median		5.1	5.3	4.8	5.1	4.7	18.0	7.0	29.0
Percentiles	25	4.5	4.7	4.1	4.5	4.1	10.0	3.0	23.0
	50	5.1	5.3	4.8	5.0	4.7	18.0	7.0	29.0
	75	5.5	6.0	5.50	5.6	5.3	28.0	11.0	36.0

TEIQue (EI) = Emotional Intelligence

Burnout subscales: *EE* Emotional Exhaustion, *DP* Depersonalization, *PA* Personal Accomplishment

Correlation analysis showed that age had a small direct correlation with self-control (*r* = 0.17, *p* = 0.05); however, there were no statistically significant correlations with other EI factors. However, there was a moderate inverse correlation between age and EE (*r* = -0.33, *p* < 0.0001) and a small inverse correlation with DP (*r* = -0.21, *p* = 0.02).

An independent-samples t-test was conducted to compare the burnout subscales and EI factors by gender and nationality. There was a statistically significant difference in global TEIQue score between men (5.1 ± 0.6) and women participants (4.9 ± 0.8)(t_110_ = 2.1, *p* = 0.04) and in the wellbeing mean score between men (5.5 ± 0.8) and women (5.1 ± 1) (t_124_ = 2.45, *p* = 0.02). In addition, the gender difference was seen in self-control score where men scored 5 ± 0.8 (women = 4.7 ± 1) with t_116_ = 2.1, *p* = 0.04. There was no statistically significant difference in burnout subscale scores between men and women. T-test demonstrated a statistically significant difference in EI factor “emotionality” among Saudis (5.2 ± 0.8) and non-Saudis (4.9 ± 0.8) (t_124_ = 2.2, *p* = 0.03), and for burnout subscales there was statistically significant difference in DP among Saudis (6.4 ± 4.8) and non-Saudis (8.5 ± 5.6) (t_124_ = -2.2, *p* = 0.03).

There was a statistically significant inverse relationship between years since graduation and EE (*r* = -0.29, p = 0.001) and DP (*r* = -0.19, *p* = 0.03) but no significant relationship with PA or EI factors. One-way ANOVA showed statistically significant differences between work duration and global TEIQue score (F4,121 = 3.25, *p* = 0.01), emotionality (F4,121 = 3.43, *p* = 0.01), EE (F4,121 = 4.7, *p* = 0.001), and DP (F4,121 = 2.6, *p* = 0.04). Those who worked for 11–15 years showed higher global TEIQue (5.4 ± 0.6, 95% CI:5.1, 5.7) and emotionality (5.5 ± 0.8, 95% CI:5.1, 5.8) scores than the other groups. Staff who worked for 5 years and less showed higher EE (24 ± 12.1, 95% CI:20, 28) and DP (8.4 ± 5.9, 95% CI:6.4,10.3) scores.

There were no statistically significant differences in EI and burnout scores among those who trained locally or abroad as well as those who held administrative positions and those who did not, or between academic and clinical staff (*p*-value > 0.05) as determined by the t-test.

Descriptive analysis among various medical specialties showed that TEIQue total score was high among participants working in medicine/pediatrics (5.28 ± 0.73) and surgery/anesthesia (5.26 ± 0.55), while EE score was high among staff in family medicine (23.2 ± 8.6) and PA score was lowest among radiology staff 20.6 ± 11.7 (Table 3).

**Table 3:** Shows demographic, TEIQue, and MBI-HSS scoring comparison between different specialties (*n* = 126)

		family medicine (*n* = 33)	medicine & pediatrics (*n* = 30)	Surgery & anesthesia (*n* = 26)	Basic science (*n* = 18)	Radiology (*n* = 10)	OB/Gyn (*n* = 5)	Dentists (*n* = 4)
Age (mean ± SD)		43.4 ± 10.4	44.7 ± 9.9	46.3 ± 7.9	48.3 ± 10.4	31.5 ± 5.6	50 ± 6.7	34 ± 9.9
Gender	Male	11	20	21	4	6	0	3
	Female	22	10	5	14	4	5	1
Nationality	Saudi	24	18	15	13	7	3	2
	Non-Saudi	9	12	11	5	3	2	2
TEIQue total score (mean ± SD)		4.9 ± .65	5.28 ± .73	5.26 ± .55	4.7 ± .98	4.8 ± .67	4.9 ± .58	4.7 ± .79
Well-being score (mean ± SD)		5.3 ± .83	5.5 ± .89	5.6 ± .81	4.9 ± 1.3	4.8 ± .73	5.1 ± .77	5.3 ± 1.0
Self-control score (mean ± SD)		4.6 ± .87	5.1 ± .89	5.1 ± .83	4.6 ± 1.1	4.7 ± .98	4.9 ± .49	4.4 ± .68
Emotionality score (mean ± SD)		5.0 ± .84	5.3 ± .87	5.1 ± .67	4.7 ± 1.0	4.9 ± .55	5.2 ± .62	4.6 ± .86
Sociability score (mean ± SD)		4.6 ± .64	5.0 ± .74	4.9 ± .88	4.5 ± .8	4.7 ± .72	4.4 ± 1.0	4.5 ± .75
MBI- Emotional Exhaustion (mean ± SD)		23.2 ± 8.6	20.2 ± 12.2	13.8 ± 7.9	19.0 ± 12.9	22.4 ± 13.1	16.8 ± 11.5	21.0 ± 12.7
MBI-Depersonalization (mean ± SD)		8.2 ± 4.8	6.6 ± 6.3	5.9 ± 4.5	7.7 ± 5.6	5.6 ± 4.7	7.0 ± 2	9.7 ± 3.6
MBI-Personal Accomplishment (mean ± SD)		31.9 ± 6.9	31.1 ± 9.5	26.6 ± 11.1	27 ± 7.6	20.6 ± 11.7	24.4 ± 7.9	26.7 ± 3.9

Burnout subscales: MBI-Emotional Exhaustion (EE = 0–54) and MBI-Depersonalization (DP = 0–30) Scores: Higher scores indicate higher degrees of burnout. MBI-Personal Accomplishment (PA = 0–48) Scores: Lower scores indicate higher degrees of burnout

TEIQue: Global Emotional Intelligence (EI) score

RII was run for burnout subscales and TEIQue items separately. RII analysis for burnout subscales (EE, DP, PA) showed that the top ranked indicator for EE was “working hard” with 3.1 ± 1.8 out of 7 and 52% relative importance points and the lowest ranked indicator was “inability to deal with difficult situations” (1.6 ± 1.6, 27%).” Being insensitive / heartless toward patients and students” was the top ranked indicator for DP (1.9 ± 1.7 out of 7, 31.5% RII) and “disregard of patient care” was the lowest ranking (0.7 ± 1.7, 11% RII). “Positively influencing others’ life via work” was top ranked PA indicator with 3.9 ± 1.6 out of 7 and 65% RII, and “feeling happy due to close interaction with clients” was the lowest ranking (2.6 ± 1.7, 43% RII) for PA.

RII values of TEIQue showed that the top three ranking indicators for EI were “I have a number of good qualities” (5.6 ± 1.4, 80% RII), “I’m pleased with my life” (5.5 ± 1.4, 79% RII), and “I believe that things will work out fine in my life” (5.4 ± 1.5, 77% RII); all indicators of wellbeing. The lowest ranked indicators were items number14, “I often find it difficult to adjust my life according to the circumstances” (2.2 ± 1.5, 35% RII), item 5 “I generally don’t find life enjoyable “ (2.3 ± 1.6, 32% RII), and item 13 “Those close to me often complain that I don’t treat them right” (2.2 ± 1.5, 31% RII). TEIQue RII details of EI indicator ranking are shown in Table 4.

**Table 4:** Descriptive analysis & relative importance analysis of the perceived indicators of emotional intelligence

TEIQue-SF items (item number in brackets)	Mean (SD)	RII	*Rank*
I feel that I have a number of good qualities (9). (WB)	5.60 (1.36)	79.9	***1***
On the whole, I’m pleased with my life (20). (WB)	5.54 (1.41)	79.1	***2***
I generally believe that things will work out fine in my life (27). (WB)	5.37 (1.52)	76.8	***3***
On the whole, I’m a highly motivated person (3)	5.29 (1.50)	75.6	***4***
I can deal effectively with people (6). (Soc)	5.28 (1.57)	75.4	***5***
I believe I’m full of personal strengths (24). (WB)	5.28 (1.32)	75.4	***6***
Generally, I’m able to adapt to new environments (29)	5.21 (1.58)	74.4	***7***
I’m usually able to find ways to control my emotions when I want to (19). (SC)	5.04 (1.48)	72.0	***8***
On the whole, I’m able to deal with stress (15). (SC)	5.02 (1.5)	71.8	***9***
Expressing my emotions with words is not a problem for me (1). (EM)	4.92 (1.63)	70.3	***10***
I would describe myself as a good negotiator (21). (Soc)	4.73 (1.45)	67.6	***11***
I’m normally able to “get into someone’s shoes” and experience their emotions (17). (EM)	4.66 (1.68)	66.6	***12***
I’m usually able to influence the way other people feel (11). (Soc)	4.63 (1.38)	66.2	***13***
Others admire me for being relaxed (30). (SC)	4.56 (1.60)	65.1	***14***
I often pause and think about my feelings (23). (EM)	4.21 (1.62)	60.2	***15***
I tend to “back down” even if I know I’m right (25). (Soc)	3.67 (1.54)	52.5	***16***
I tend to get involved in things I later wish I could get out of (22). (SC)	3.64 (1.52)	52.0	***17***
On the whole, I have a gloomy perspective on most things (12). (WB)	3.52 (1.88)	50.3	***18***
I don’t seem to have any power at all over other people’s feelings (26). (Soc)	3.38 (1.53)	48.3	***19***
I often find it difficult to stand up for my rights (10). (Soc)	3.22 (1.63)	46.0	***20***
I tend to change my mind frequently (7). (SC)	2.97 (1.57)	42.4	***21***
I often find it difficult to show my affection to those close to me (16). (EM)	2.95 (1.63)	42.2	***22***
I usually find it difficult to regulate my emotions (4). (SC)	2.91 (1.61)	41.6	***23***
I often find it difficult to see things from another person’s viewpoint (2). (EM)	2.83 (1.57)	40.5	***24***
I normally find it difficult to keep myself motivated (18)	2.83 (1.61)	40.5	***25***
Many times, I can’t figure out what emotion I'm feeling (8). (EM)	2.73 (1.55)	39.0	***26***
I find it difficult to bond well even with those close to me (28). (EM)	2.54 (1.63)	36.3	***27***
I often find it difficult to adjust my life according to the circumstances (14)	2.46 (1.32)	35.1	***28***
I generally don’t find life enjoyable (5). (WB)	2.27 (1.56)	32.4	***29***
Those close to me often complain that I don’t treat them right (13). (EM)	2.17 (1.53)	31.1	***30***

*EM* emotionality, *SC* self-control, *Soc* sociability, *WB* well-being

TEIQue-SF items reproduced with permission by the copyright holder

© K. V. Petrides 1998 –. All rights reserved. http://www.psychometriclab.com

**Fig. 1:**
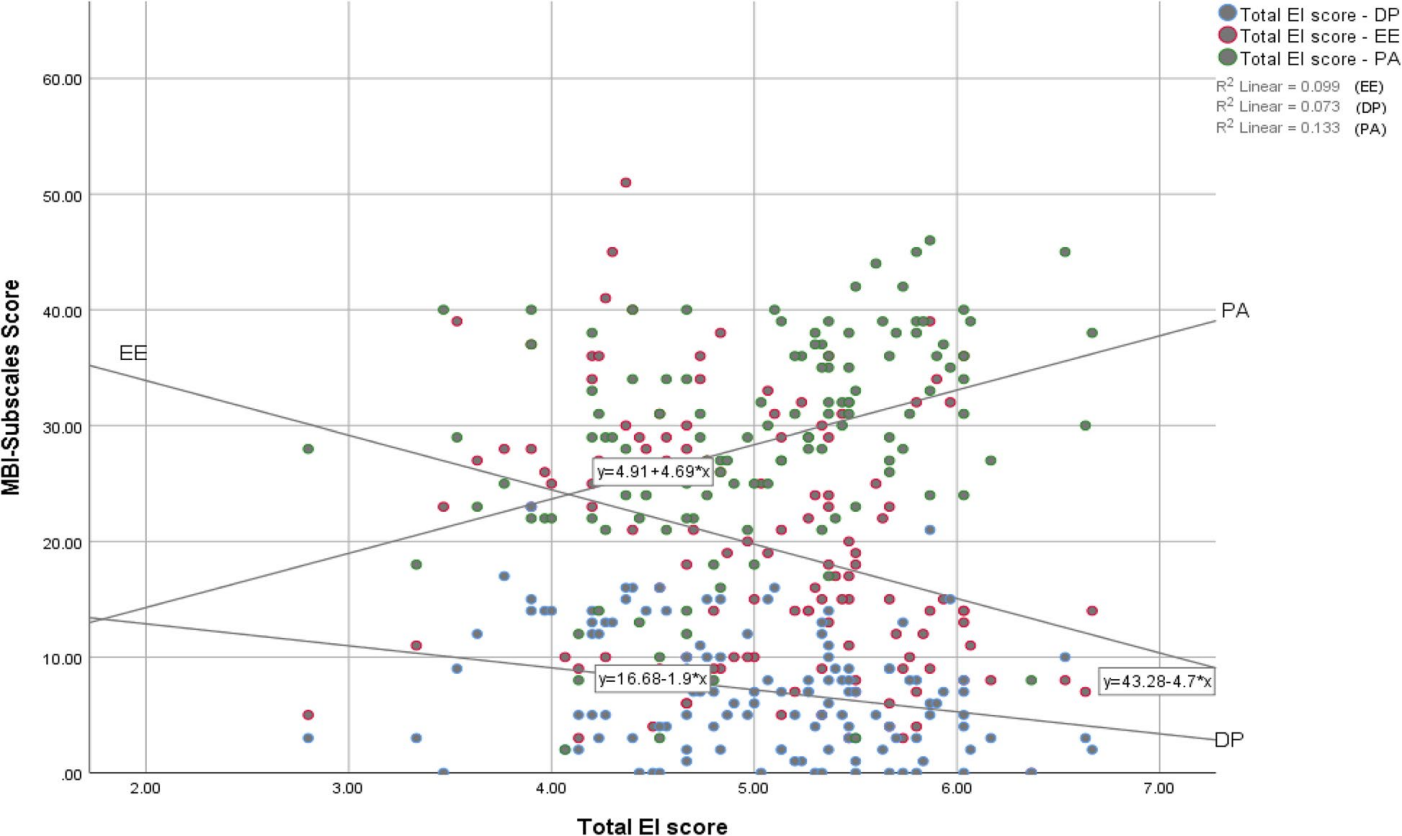
Scattered graph showing the relationship between burnout subscales (DP, EE, PA) and total emotional intelligence score

A bivariate Pearson's (r) test of correlation showed a strong positive inter-factorial correlation among global trait EI and its factors with *p* = 0.01. Burnout subscales EE and DP had a strong positive correlation (*r* = 0.63, *p* = 0.01), EE and PA had a weak positive correlation (*r* = 0.2, *p* = 0.05), and DP and PA showed no correlation. Moderate and weak negative correlations were obtained between EI factors and burnout subscales (EE, PD). Moderate positive correlations were obtained between global TEIQue, EI and its factors (well-being, self-control, emotionality) with burnout subscale PA, but no correlation was obtained between sociability and PA (Table 5, Fig. 1).

**Table 5:** Demonstrating the relationship between EI factors and burnout subscales

	1	2	3	4	5	6	7	8
1-Global EI score	1							
2-Well-being	.846^b^	1						
3-Self-control	.839^b^	.622^b^	1					
4-Emotionality	.838^b^	.618^b^	.596^b^	1				
5-Sociability	.768^b^	.532^b^	.601^b^	.532^b^	1			
6-EE	-.314^b^	-.231^b^	-.301^b^	-.211^a^	-.302^b^	1		
7-DP	-.270^b^	-.212^a^	-.188^a^	-.288^b^	-.206^a^	.634^b^	1	
8-PA	.365^b^	.420^b^	.264^b^	.364^b^	.127	.197^a^	.063	1

Burnout subscales *EE* Emotional Exhaustion, *DP* Depersonalization, *PA* Personal Accomplishment

^a^Correlation is significant at the 0.05 level (2-tailed)

^b^Correlation is significant at the 0.01 level (2-tailed)

A multiple regression analysis was run to predict EE, DP, and PA based on gender, age, nationality, field of specialty, academic position, clinical position, work duration, and Total TEIQue scores. These variables statistically significantly predicted EE [*F*(8,117) = 5.088, *p* < 0.0005, R^2^ = 0.258]; DP, [*F*(8,117) = 2.585, *p* = 0.012, R^2^ = 0.150]; and PA, [*F*(8,117) = 4.134, *p* < 0.0005, R^2^ = 0.220]. The field of specialty, work duration, and total TEIQue score added statistically significantly to the prediction of EE at *p* ≤ 0.05, DP at *p* ≤ 0.005, and PA at *p* ≤ 0.005 (Table 6).

**Table 6:** A multiple regression analysis to predict EE, DP, and PA based on gender, age, nationality, field of specialty, academic/clinical position, work duration, and total EI score

Coefficients							
	Unstandardized Coefficients		Unstandardized Coefficients	t	Sig	95.0% Confidence Interval for B	
	B	Std. Error	Beta			Lower Bound	Upper Bound
Dependent Variable: EE							
(Constant)	54.281	9.316		5.827	.000	35.831	72.731
Total EI score	-4.287	1.249	-.287	-3.433	.001	-6.761	-1.814
Gender	.703	1.884	.032	.373	.710	-3.028	4.434
Age	-.084	.155	-.078	-.542	.589	-.391	.223
Nationality	-2.177	2.268	-.095	-.960	.339	-6.668	2.315
Field of specialty	-1.273	.554	-.186	-2.298	.023	-2.370	-.176
Academic position	.605	.671	.084	.902	.369	-.723	1.934
Clinical position	.020	1.213	.002	.016	.987	-2.383	2.423
Work duration	-2.174	1.099	-.308	-1.977	.050	-4.351	.003
Dependent Variable: DP							
(Constant)	19.43	4.693		4.141	.000	10.138	28.728
Total EI score	-1.796	.629	-.255	-2.85	.005	-3.042	-.550
Gender	-.379	.949	-.037	-.400	.690	-2.259	1.500
Age	-.082	.078	-.161	-1.05	.297	-.237	.073
Nationality	1.604	1.143	.148	1.404	.163	-.659	3.867
Field of specialty	-.324	.279	-.100	-1.16	.248	-.877	.229
Academic position	.196	.338	.058	.581	.562	-.473	.866
Clinical position	-.421	.611	-.067	-.688	.493	-1.631	.790
Work duration	-.134	.554	-.040	-.242	.809	-1.231	.963
Dependent Variable: PA							
(Constant)	7.028	8.212		.856	.394	-9.236	23.291
Total EI score	4.568	1.101	.355	4.149	.000	2.388	6.748
Gender	.867	1.661	.046	.522	.602	-2.421	4.156
Age	-.016	.137	-.018	-.120	.905	-.287	.254
Nationality	1.459	1.999	.074	.730	.467	-2.500	5.418
Field of specialty	-1.577	.488	-.268	-3.229	.002	-2.544	-.610
Academic position	-.283	.591	-.046	-.478	.633	-1.454	.888
Clinical position	1.371	1.070	.120	1.282	.202	-.747	3.489
Work duration	-.318	.969	-.052	-.329	.743	-2.238	1.601

Predictors: (Constant), Total EI score, gender, age, nationality, field of specialty, academic/clinical position, work duration

Burnout subscales *EE* Emotional Exhaustion, *DP* Depersonalization, *PA* Personal Accomplishment

TEIQue Total score of emotional intelligence

TEIQue as a dependent variable in the prediction model based on gender, age, nationality, field of specialty, academic position, clinical position, and work duration showed *F*(7,118) = 1.67, *p* = 0.12, R^2^ = 0.09. These variables did not statistically significantly predict TEIQue at *p* > 0.05.

## Discussion

In this study, we reported total and subscales scores of burnout which were within the range reported in previous literature [9]. Contrarily, our findings for EE and PA subscales were lower than those reported by Maslach among the normative medical provider population [21]. The DP score among our cohort was similar to the MBI Maslach normative DP score [21]. These similarities and differences may be related to differences in workplace cultures. Schaufeli and colleagues reported lower MBI scores among approximately four thousand healthcare workers "normative Dutch sample," and referred to these cultural differences [25, 32]. Hence, our result may also be affected by our study cohort's structure, and sampling and reporting bias cannot be ruled out. It may be speculated that those who agreed to participate in the study may have felt less stress to be enrolled in the study and may be considered healthy employees [25].

Regarding EI and its four factors, we reported results that were similar to the normative sample of the instrument developer [22]. A higher EI and its elements scores were reported to correspond with coping and adaptation to stressful events [34]. An inverse interrelation was reported between burnout and EI [16, 17]. This relationship was found to be small to moderate in our cohort group as the reported higher EI scores were correlated with lower burnout scores. Our cohort perceived wellbeing items as essential indicators for better EI. These findings are in agreement with Schutte et al.’s study that found individuals with higher EI having higher emotional wellbeing and self-esteem [35].

Feeling that the job drains one's energy, as reflected by the item "working hard and feeling drained by the job," was an indicator of the EE subscale of burnout. While feeling insensitive towards others, as stated by the item, "feeling callous and hard-hearted," was an essential indicator of DP and being skeptical about the purpose of one's job. Contrarily, "having a positive effect on others' lives" was the most important indicator of PA burnout subscales, and this reflects the core of the health care professionals by leaving a positive effect on other people’s, students’, or patient's life. West et al. reported that feeling burned out was the single item that can indicate EE, and becoming callous to patients was the single item that showed DP [36]. In our opinion, feeling drained by the job was the most critical indicator for burnout; for example, when a physician's energy is drained by long working hours, he becomes insensitive to others, and this feeling which contradicts the core of his profession, causes him to underestimate his professional and human achievements.

We reported a significant relationship between burnout and EI with age, gender, and nationality. A similar age range in other studies was found to be inversely related to burnout [37]; however, we could not identify any relationship between age and EI factors apart from a slight direct connection with the self-control factor. In contrast with other studies [28, 38], we reported EI and its elements higher in men than in women, which can be explained based on cultural differences [39]. It was reported that older women with job control and satisfaction have higher EI [28]. The observed gender differences by other studies in EI were negligible [22, 28]. Thus, the practice of EI in day-to-day work of physicians and academic educators is required at different levels of responsibilities, with particular emphasis on those in leadership positions [40]. Among our cohort, DP was found to be higher among non-Saudis; Leiter and Maslach described a continuum between engagement and burnout and identified DP (cynicism) as more important than EE (exhaustion) in the burnout continuum profile [31] compared to native physicians, foreign physicians reported higher burnout and lack of professional support [41]. High scores on cynicism (DP) and exhaustion (EE) were observed among those who newly joined the institution.

We found that EI was the common variable that contributed to the prediction of burnout subscales (EE, DP, PA). By contrast, work duration and field of specialty contributed to predicting EE besides EI. Additionally, field of specialty contributed to the prediction of PA. Individual demographic variables are not considered predictors for burnout; work-life variables can escalate or deescalate burnout direction [42]. Six domains have been identified as workplace risk factors for burnout: workload, job control, supportive environment, recognition and rewards, equitability, and organizational values [42]. We reported variations among different medical departments in the EI and burnout scores. Staff working in departments with high EI (and its factors) and with low EE and DP scores may suggest the stability of the department. Contrarily staff of departments with lower EI and its factors and high EE and DP scores may indicate the need for employees for coworker support to defeat burnout and maintain their well-being and ultimately preserve patient safety and quality of health care. We found that staff in the family medicine department scored higher on EE and DP with lower EI scores; a ten-year follow-up study of primary care physicians found that patients' frequent contact and demands led to burnout [43]. unairness and favoritism have also been reported as major problem that put departments in crisis leading to burnout [42]. Staff in the surgery department scored lower EE and PD and higher EI compared with other departments; this result is contrary to what was reported in the meta-analysis that high burnout EE or PD scores exist in approximately 1:3 surgeons with severity varying among specialties [44, 45].

### Limitations

This study is not without limitations. It is a questionnaire-based study and thus has the limitations associated with questionnaires, including difficulty in conveying the emotional elements; responders’ hidden agenda, lack of personalization, and difficulty in understanding and interpreting questions. We used standardized questionnaires that are widely used in research, and accordingly interpreted our results. The number of participants in burnout studies of healthcare workers is usually low [45]. Heijden reported several reasons for not responding to a burnout survey, including lack of time and energy and, length of the questionnaires [6]. These reasons may point to burnout among those who did not respond to the survey and raise the need for screening using two questions validated for burnout detection [36]. Another limitation of our study is not including other factors that may contribute to burnout and the effect of cultural differences. Therefore, we recommend a longitudinal study of those at risk of being exposed to burnout because of the nature of their job or their culture. Moreover, using more than one instrument may help identify those with positive- negative/negative–positive results.

## Conclusions

This study illustrates an inverse relationship between burnout and emotional intelligence scores among medical academic educators and clinicians. It proposes the need for taking measures and implementing a system for early detection of burnout in staff, and providing support to enhance emotional intelligence and requisite care.

## Data Availability

The datasets used and analyzed during this study are available from the corresponding author on reasonable request.

## Abbreviations

EE: Emotional exhaustion
DP: Depersonalization
PA: Personal accomplishment
MBI-HSS: Maslach Burnout Inventory- Human Services Survey
TEIQ-SF: The Trait Emotional Intelligence Questionnaire- Short Form
IRB: Institutional Research Board
aMBI: The Abbreviated Maslach Burnout Inventory
EI: Emotional Intelligence
